# The Associations of Grey Matter Thickness and White Matter Parameters on Corticospinal Excitability in Adolescence: A Navigated TMS Study

**DOI:** 10.1007/s10548-026-01236-0

**Published:** 2026-07-18

**Authors:** Ella Kinnunen, Laura Säisänen, Heidi Gröhn, Eero A. Haapala, Timo A. Lakka, Sara Määttä, Elisa Kallioniemi

**Affiliations:** 1 Institute of Clinical Medicine, Clinical Neurophysiology, University of Eastern Finland, Kuopio, Finland; 2 Institute of Clinical Medicine, Neurology, University of Eastern Finland, Kuopio, Finland; 3 Neuro center, Neurology, Kuopio University Hospital, Kuopio, Finland; 4 Diagnostic Imaging Center, Kuopio University Hospital, Kuopio, Finland; 5 Active Life Laboratory, Preventive Health Research Unit, South-Eastern Finland University of Applied Sciences, Mikkeli, Finland; 6 Institute of Biomedicine, School of Medicine, University of Eastern Finland, Kuopio, Finland; 7 Faculty of Sport and Health Sciences, University of Jyväskylä, Jyväskylä, Finland; 8 Department of Clinical Physiology and Nuclear Medicine, Kuopio University Hospital, Kuopio, Finland; 9 Kuopio Research Institute of Exercise Medicine, Kuopio, Finland; 10 Department of Clinical Neurophysiology, Kuopio University Hospital, Kuopio, Finland; 11 Department of Biomedical Engineering, New Jersey Institute of Technology, Newark, NJ USA

**Keywords:** Diffusion tensor imaging, Transcranial magnetic stimulation, Motor cortex, Cortical excitation, Adolescent

## Abstract

The relationship between brain structure and functional measures, such as resting motor threshold (rMT), is of interest and has been studied in adults, but there is a lack of research in adolescents. We studied the correlation between rMT and the grey matter thickness as well as white matter parameters of the motor cortex, using magnetic resonance imaging and navigated transcranial magnetic stimulation. Forty-five participants (twenty boys and twenty-five girls) aged sixteen to nineteen years were recruited for the study. We determined rMT for the abductor pollicis brevis muscle and investigated the correlation between rMT and grey matter thickness and white matter parameters, fractional anisotropy (FA), mean diffusivity (MD), radial diffusivity (RD) and axial diffusivity (AD) in the hand knob and in the extended hand knob. Grey matter thickness in the primary motor cortex was associated with rMT. We also discovered that white matter parameters RD and MD in a broader area of the primary motor cortex were positively associated with rMT. Both grey and white matter appear to influence cortical excitability in adolescence. Understanding the normal development of the brain is necessary to understand pathological neurodevelopmental processes.

**Supplementary Information:**

The online version contains supplementary material available at https://doi.org/10.1007/s10548-026-01236-0.

## Introduction

Adolescence is a critical period for brain development with diffuse structural and functional changes that enable maturation of cognition and motor functions (Arain et al. 2013). While the gross structural changes throughout adolescent brain development have been explained (Blakemore 2012), little is known of the functional consequences of these developmental changes, particularly within the motor system. Transcranial magnetic stimulation (TMS) can provide an important measure of cortical excitability in a non-invasive manner, whereas magnetic resonance imaging (MRI) allows sophisticated structural characterization of the brain.

MRI can be used to study the brain structures of adolescents safely (Holland et al. 2014). On a macroscale, MRI studies have shown that total cerebral white matter volume increases linearly throughout adolescence (Giedd 2004; Sowell et al. 2002), whereas grey matter volume follows an inverted U-shaped pattern (Giedd 2004). An increase in white matter volume has been suggested to result from axonal myelination (Giedd 2004) and a decrease in grey matter volume at least partially from synaptic pruning (Gogtay et al. 2004). The motor cortex and primary sensory areas undergo faster maturation than, for example, the prefrontal cortex (Gogtay et al. 2004), and they may reach full maturity by the age of 10 years (Wu et al. 2014). However, maturation of the superficial white matter continues at least to age 18 in those brain areas (Wu et al. 2014).

Diffusion tensor imaging (DTI) is an MRI technique that measures directional water molecule diffusion, and it is suitable for studying white matter structures (Assaf & Pasternak 2008; Le Bihan et al. 2001). DTI provides measures such as fractional anisotropy (FA), mean diffusivity (MD), radial diffusivity (RD) and axial diffusivity (AD). FA describes directionally dependent diffusion, reflects the white matter fiber density and axonal diameter and is used to measure white matter integrity (Beaulieu 2002; Fjell et al. 2008). MD reflects overall diffusion and increased MD indicates decreased white matter integrity. RD reflects diffusivity perpendicular to fiber tracts and the increases in RD result from demyelination (Song et al. 2005). AD describes diffusion parallel to fiber tracts and a decrease in AD may be an indication of axonal damage (Song et al. 2003). There is evidence that MD, RD and AD decrease during maturation in multiple brain areas (Kumar et al. 2012; Qiu et al. 2008). Conversely, studies have shown that FA increases during maturation between childhood and adulthood in multiple brain regions such as internal capsule and basal ganglia (Barnea-Goraly et al. 2005; Schmithorst and Yuan 2010).

TMS is a noninvasive method for examining the function of the central nervous system. When the TMS pulse is targeted to the primary motor cortex at a suprathreshold intensity, it produces a motor evoked potential (MEP) that can be recorded from the target muscle (Kobayashi and Pascual-Leone 2003). Motor threshold (MT) describes the minimal intensity of a TMS-pulse to cause a motor response in the target muscle in 50% of the stimulations (Rossini et al. 1999). When MT is assessed from a relaxed target muscle, it is called resting MT (rMT) (Rossini et al. 2015). rMT assesses corticospinal excitability and is influenced by glutamatergic neurotransmitter systems and agents that block voltage-gated sodium channels (Vucic et al. 2023). Based on previous studies, various factors affect rMT. For example, rMT decreases during development from childhood to adolescence (Säisänen et al. 2018) and increasing coil-to-cortex distance (CCD) increases rMT (Herbsman et al. 2009; List et al. 2013; Rosso et al. 2017). Moreover, white matter fiber orientation in cortico-pyramidal tracts also affects rMT (Herbsman et al. 2009). In older adults aged 50–75 years, rMT has been correlated positively with cortical thickness (List et al. 2013). In addition, rMT of the dominant hand has been correlated negatively with the grey matter volume of the dominant hemisphere in adults aged 22–65 years (Rosso et al. 2017). rMT has also been found to correlate negatively with regional FA values (Klöppel et al. 2008).

Despite the existing evidence in adults, correlations of structural measures of white and grey matter in the motor cortex with functional measures, such as rMT, especially in adolescence, remain unknown. It is essential to study how brain structures are related to cortical excitability during adolescence in an effort to delineate normal neurodevelopment. Understanding the normal neurodevelopment is necessary for explaining causes of psychiatric disorders with pathological neurodevelopmental origin occurring in adolescence (Miguel-Hidalgo 2013; Paus et al. 2008). Therefore, we investigated normal adolescent brain development by evaluating the associations of grey and white matter structures with cortical excitability in healthy adolescents.

## Materials and Methods

### Participants

Twenty boys and twenty-five girls, all right-handed, aged sixteen to nineteen years (range 16.3–19.1) were recruited from a population sample of 277 adolescents who attended the 8-year follow-up examinations of the Physical Activity and Nutrition in Children (PANIC) study conducted at the Institute of Biomedicine, the University of Eastern Finland (Eloranta et al. 2012). Handedness of the participants was assessed with Waterloo handedness questionnaire (Steenhuis et al. 1990) and all included participants were right-handed. Exclusion criteria included common contraindications to TMS and MRI (Rossi et al. 2009), using medications affecting the central nervous system and known psychiatric or neurological disorders. Participants were informed about the study and procedures. All participants provided written informed consent. The study was approved by the Research Ethics Committee of the Hospital District of Northern Savo (366/2017). The study was carried out in accordance with the principles of the Declaration of Helsinki as revised in 2008.

### Magnetic Resonance Imaging

MRI was performed using a 3.0 T MRI-scanner (Philips Achieva 3.0 T X, Philips, Netherlands) for a whole-brain field of view. Anatomical MRI included three-dimensional (3D) T1-weighted, T2-weighted and FLAIR sequences. In addition to anatomical MRI, DTI was performed (slice thickness 2 mm, in-plane resolution 2 mm × 2 mm, 16 unique diffusion directions at b0 = 1000 or 800 s/mm2).

T1-weighted images (TR 8.07 ms, TE 3.7 ms, flip angle 8°, 1 × 1 × 1 mm^3^ resolution) were used for neuronavigation in TMS (nTMS). They were also used in grey and white matter analyses. Before proceeding to the next section of the study, a neuroradiologist screened the images to exclude any structural abnormalities.

### Navigated TMS

The eXimia stimulator (version 3.2.2) and biphasic coil were used in nTMS (Nexstim Plc., Helsinki, Finland). The abductor pollicis brevis (APB) muscle was identified and disposable Ag–AgCl surface electrodes were placed on the muscle for recording MEPs using the belly-tendon montage. Throughout the measurement, muscle activity was monitored online and recorded by stimulus-locked EMG (Nexstim Plc., Helsinki, Finland). Stimulation, and thus determination of the optimal representation site, i.e., hotspot, and rMT, was performed on both hemispheres, and the starting hemisphere was randomized. Hotspot identification was initiated at an intensity of 50% of the maximum stimulator output (MSO), and the stimulation intensity was systematically adjusted to yield MEPs with amplitudes in the range of approximately 200–1000 µV.

**Fig. 1:**
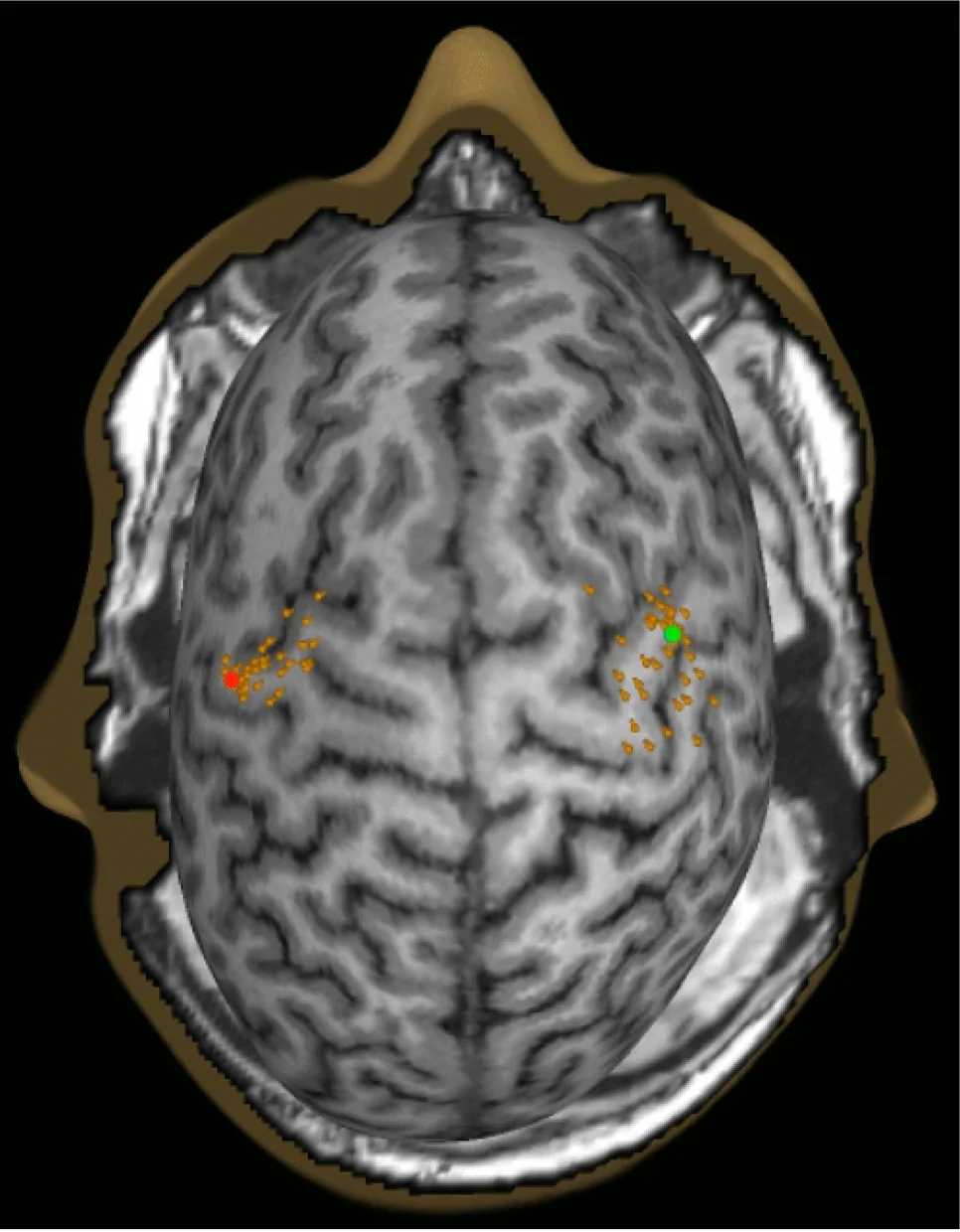
An example of locations that evoked motor evoked potentials (MEPs) in the abductor pollicis brevis muscle with an amplitude of 50 µV or over while determining the optimal representation site, i.e., the hotspot on both hemispheres. The stimulation sites are indicated by the orange dots. The stimulation sites represent the search for the hotspot and not a systematic motor map. The hotspot on the left hemisphere is indicated in red, on the right hemisphere in green. In this participant, the resting motor threshold (rMT) on the left hemisphere was 36 percent of the maximum stimulator output (%-MSO) and 34%-MSO on the right hemisphere. The mapping intensity calculated retrospectively was between 120–125% of the rMT

The hotspot was first specified as the stimulation site where the largest MEP amplitudes were repeatedly obtained (Fig. 1). While determining the hotspot, the coil orientation was perpendicular to the nearest sulcus and the navigation software indicated optimal coil tilting. At the hotspot, coil orientation was rotated ± 90° to find the optimal electric field direction for the final target. rMT was determined at the hotspot in both hemispheres by using the threshold hunting paradigm, Motor Threshold Assessment Tool (MTAT) (Awiszus and Borckardt 2012) as a percentage of the maximum stimulator output (%-MSO) with an MEP amplitude limit of 50 µV until the confidence interval was 95%. EMG was used to monitor the relaxation of the examined muscles. Peak-to-peak analyses were used to determine the MEP amplitude. Coil-to-cortex distance (CCD) was measured using the navigation software as the peeling depth from the scalp to the surface of the cortex.

### MRI Data Analysis

**Fig. 2:**
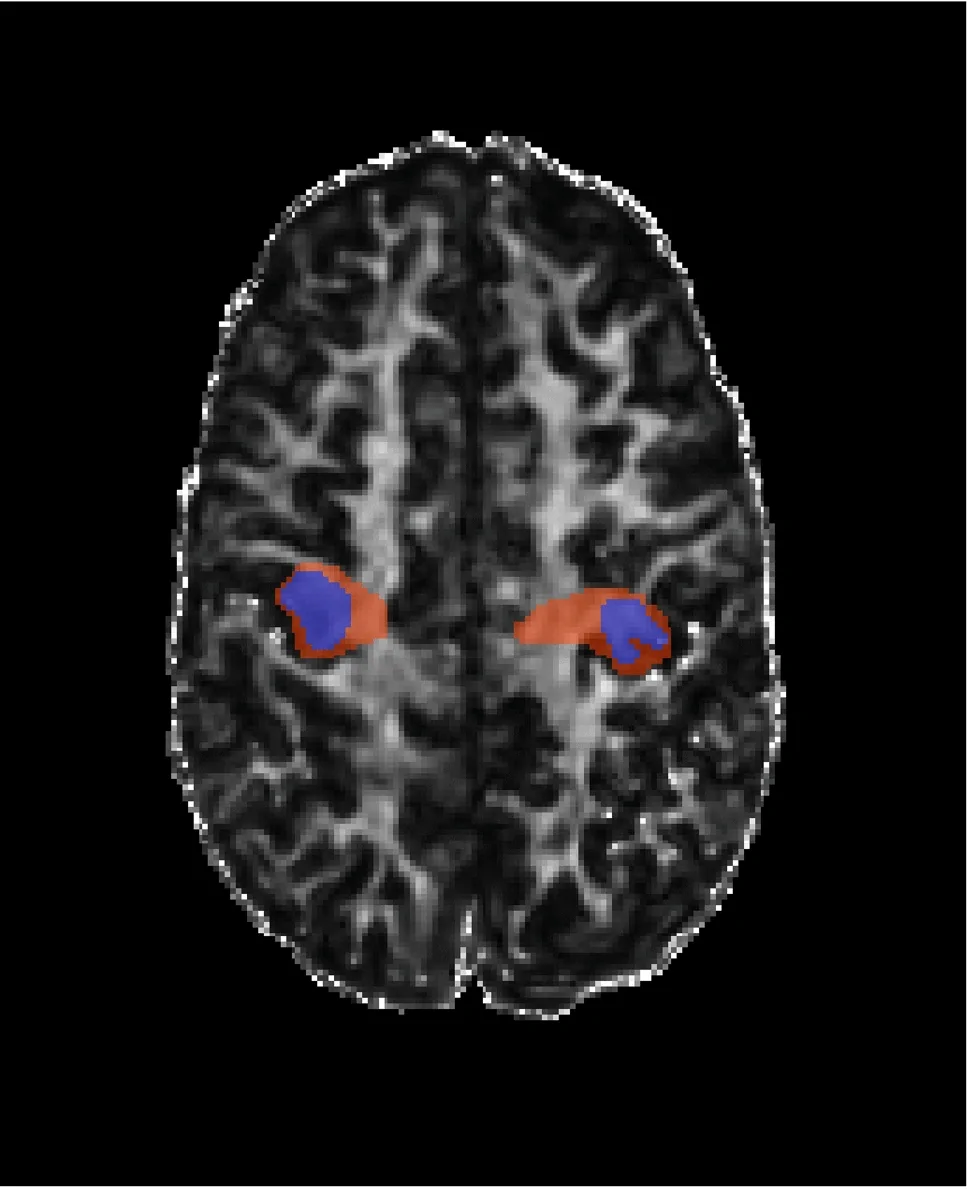
An example of drawn regions of interest (ROIs) on both hemispheres is shown in the FA-image. The hand knob area is shown in blue, the extended hand knob area in red

FA, MD, RD, and AD images were created by fitting a tensor model to the raw diffusion data using FDT from the FSL package (Smith et al. 2004). Diffusion images were registered with T1 data, and regions of interest (ROIs) placed on the hand knob and extended hand knob areas were manually drawn on the diffusion-weighted images, with the help of T1-weighted images. ROIs were drawn on the slice in which both left and right-hand knob areas were representative. The hand knob area was extended to medial and posterior direction to define the extended hand knob area (Fig. 2).

The ROIs were also applied to measure the mean thickness of the grey matter in the left and the right hand knobs using the Freesurfer software (Version 6.0, Boston, USA) (Fischl 2012). The analysis comprised motion correction, skull stripping, segmentation of subcortical white matter and deep grey matter structures, intensity normalization, determination of the pial surface, as well as the grey/white matter boundary, and topology correction. After this automated process, the tissue borders for each individual were visually inspected and, where necessary, manually corrected before rerunning the subsequent steps. Finally, the mean cortical thickness for the ROIs was derived from the resulting cortical reconstruction. Two subjects were excluded from the study due to artefacts or technical issues with the MRI images.

### Statistical Analysis

The statistical analyses were performed by SPSS version 27 (IBM Corporation, Somers, NY, USA). Grey matter thickness and white matter parameters (FA, MD, RD, AD) were determined from the hand knob and the extended hand knob area on both hemispheres, rMT was specified for the right and left hemisphere. The independent-samples t-test was used to study the differences between boys and girls. We used Pearson’s correlation coefficients to examine the correlation between rMT and CCD as well as the correlations of age with rMT, grey matter thickness and white matter parameters. Correlations between rMT, grey matter thickness and white matter parameters were studied for both hemispheres separately using partial correlation coefficients adjusted for sex, age and CCD. As the correlations were fairly similar between hemispheres based on Pearson’s correlation coefficients (Supplementary Table 1) and we made the assumption about similar correlations, data from right and left hemispheres were combined for statistical analyses. A linear mixed-effects model was used with rMT as the dependent variable, sex and participant as factors and grey matter thickness, white matter parameters, age and CCD as covariates. After that, we used a linear mixed-effects model to verify the similar correlations between both hemispheres by using the interaction effects for all parameters separately (grey matter thickness, FA, MD, RD, AD), and the coefficients were close to equal in all comparisons. A P-value < 0.05 was considered statistically significant.

## Results

The final number of subjects was 41 after excluding participants due to technical issues with the MRI images (n = 2) and structural abnormalities (n = 2).

TMS and MRI were both well tolerated by all participants, and no side effects were observed. The mean rMT was 41.8%-MSO (SD 7.5) on the left hemisphere and 42.3%-MSO (SD 7.1) on the right hemisphere. The mean values of rMT, CCD, grey matter thickness and white matter parameters are shown in Table 1.

**Table 1:** The values of resting motor threshold (rMT) expressed as a percentage of the maximal stimulator output, coil-to-cortex-distance (CCD), grey matter (GM) thickness, fractional anisotropy (FA), mean diffusivity (MD), radial diffusivity (RD) and axial diffusivity (AD) are presented for both left and right hemispheres. The values of GM thickness and white matter parameters FA, MD, RD, and AD are presented for the hand knob and the extended hand knob. Presented as mean (SD)

		Girls (n = 23)	Boys (n = 18)
Age (years)		17.8 (0.8)	18.0 (0.8)
rMT (-%MSO)	Left	40.4 (8.3)	43.7 (6.0)
	Right	40.9 (8.0)	44.2 (5.5)
CCD (mm)	Left	12.1 (1.5)	12.9 (1.5)
	Right	12.6 (1.6)	14.2 (1.9)
GM thickness, hand knob (mm)	Left	2.5 (0.38)	2.5 (0.20)
	Right	2.6 (0.32)	2.4 (0.26)
GM thickness, extended hand knob (mm)	Left	2.4 (0.29)	2.5 (0.13)
	Right	2.4 (0.22)	2.3 (0.24)
FA hand knob	Left	0.43 (0.061)	0.44 (0.059)
	Right	0.37 (0.052)	0.39 (0.057)
MD hand knob (× 10^–3^ mm^2^/s)	Left	0.68 (0.038)	0.70 (0.034)
	Right	0.77 (0.040)	0.77 (0.054)
RD hand knob (× 10^–3^ mm^2^/s)	Left	0.51 (0.052)	0.51 (0.047)
	Right	0.60 (0.048)	0.59 (0.060)
AD hand knob (× 10^–3^ mm^2^/s)	Left	1.0 (0.060)	1.1 (0.074)
	Right	1.1 (0.069)	1.1 (0.089)
FA extended hand knob	Left	0.37 (0.035)	0.38 (0.035)
	Right	0.36 (0.031)	0.37 (0.039)
MD extended hand knob (× 10^–3^ mm^2^/s)	Left	0.77 (0.057)	0.77 (0.038)
	Right	0.83 (0.049)	0.82 (0.050)
RD extended hand knob (× 10^–3^ mm^2^/s)	Left	0.62 (0.063)	0.61 (0.036)
	Right	0.67 (0.054)	0.66 (0.052)
AD extended hand knob (× 10^–3^ mm^2^/s)	Left	1.1 (0.056)	1.1 (0.063)
	Right	1.1 (0.056)	1.1 (0.076)

### The Influence of Coil-to-Cortex Distance (CCD), Age and Sex

CCD was positively associated with rMT on both the left hemisphere (r = 0.48, P = 0.003) and the right hemisphere (r = 0.53, P = 0.001). rMT was not associated with age on the left (r = -0.14, P = 0.38) or the right hemisphere (r = -0.23, P = 0.16). Age was not associated with grey matter thickness of the hand knob area on the left hemisphere (r = -0.17, P = 0.50) or the right hemisphere (r = -0.22, P = 0.16). Age was associated with white matter parameters MD (r = -0.48, P = 0.002), RD (r = -0.38, P = 0.013) and AD (r = -0.32, P = 0.040) of the hand knob area on the right hemisphere. On the right hemisphere, there was no association between age and FA of the hand knob area, nor MD, RD, AD, or FA in the extended hand knob area. On the left hemisphere, age was not associated with MD, RD, AD or FA of the hand knob or the extended hand knob area parameters. Boys had a higher CCD on the right hemisphere than girls (F = 1.09, Md = 1.60, 95% CI = 0.45 to 2.7, P = 0.008) and a greater total amount of the grey matter (F = 1.49, Md = 102.4, 95% CI 63.7 to 141.1, P < 0.001) and white matter (F = 2.61, Md 60.4, 95% CI = 31.7 to 89.1, P < 0.001).

### Grey Matter

There was a positive correlation between rMT and grey matter thickness of the hand knob area (R = 0.30, P = 0.018). The correlation between rMT and grey matter thickness of the extended hand knob area was not statistically significant (P = 0.53).

### White Matter

**Fig. 3:**
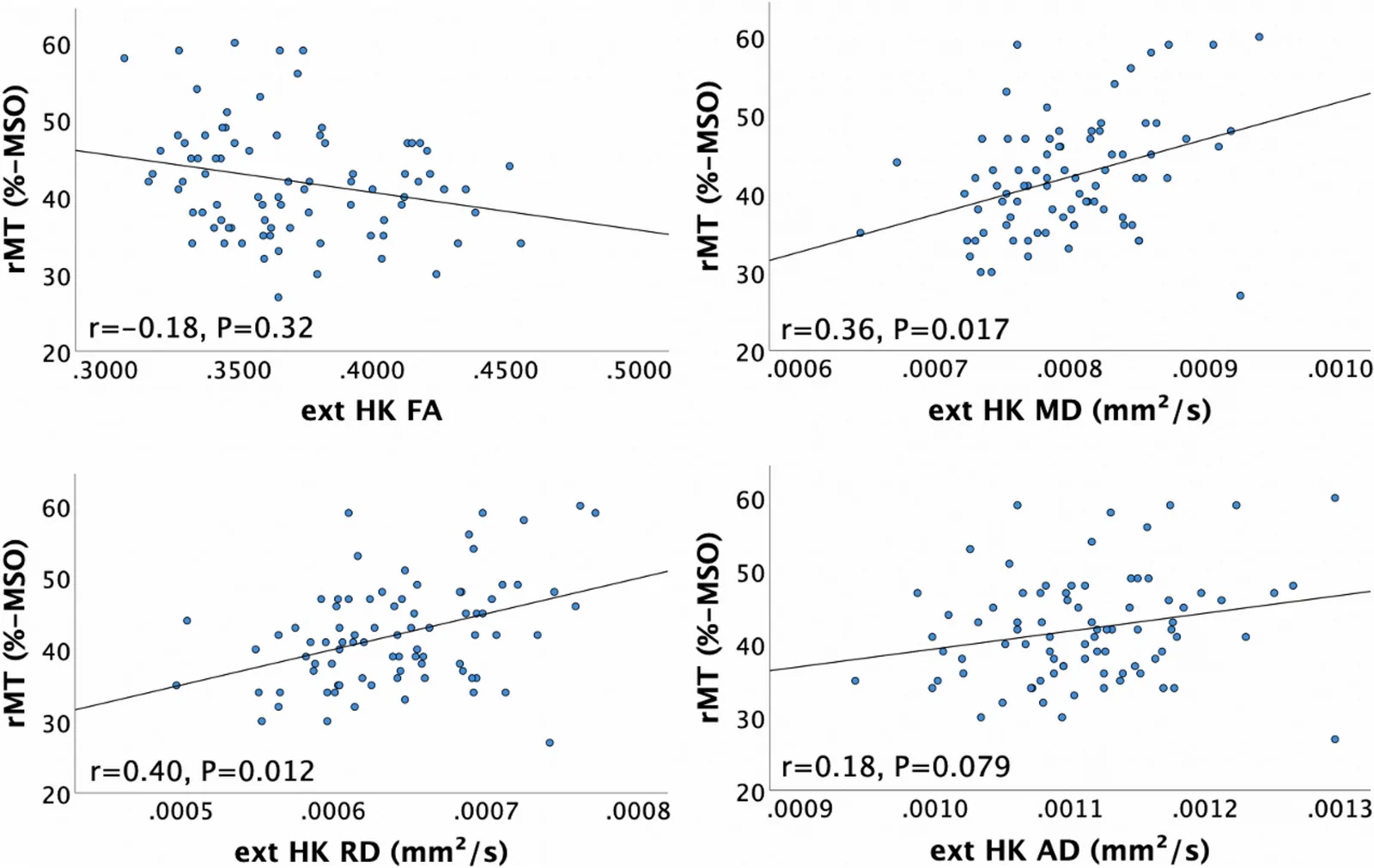
Relationship between resting motor threshold (rMT) and white matter parameters radial diffusivity (RD), mean diffusivity (MD), fractional anisotropy (FA) and axial diffusivity (AD) of the extended hand knob area. In scatter plots, the extended hand knob is referred to as ext HK

rMT correlated positively with RD of the extended hand knob (r = 0.34, P = 0.028) and MD of the extended hand knob area (r = 0.31, P = 0.043). Corresponding correlations were not statistically significant for the hand knob area. rMT was not statistically significantly correlated to FA or AD on either the extended hand knob or the hand knob (Fig. 3).

## Discussion

We found that rMT correlated positively with the grey matter thickness in the hand knob area in healthy adolescents. We also found a positive correlation between rMT and white matter variables RD and MD in the extended hand knob area. On the contrary, we did not find a correlation between rMT and FA in either the hand knob area or the extended hand knob area.

There are no previous studies on the correlation between cortical excitability and brain structures in adolescence. We observed that a thicker cortex was associated with higher rMT, thus lower cortical excitability. There is no previous knowledge concerning adolescents, but similarly to our results in older adults aged 50–75 years, a higher rMT correlated with higher cortical thickness of the hand knob (List et al. 2013). In contrast, rMT was not associated with cortical thickness in healthy older individuals with a mean age of 72 years (Niskanen et al. 2011). Furthermore, rMT correlated inversely with grey matter volume of the dominant hemisphere in healthy adults aged 22–65 years (Rosso et al. 2017). However, Rosso et al. used a different methodology, as they used grey matter volume in the analysis instead of grey matter thickness. A thin cortex may be very folded, leading to a substantial grey matter volume, possibly explaining the differences between the results. There is no corresponding study in young and middle-aged adults (roughly 25–60 years) conducted with a methodology similar to ours. Therefore, it is unclear whether the association between rMT and cortical thickness would be similar to what we found in adolescence, or whether adolescents and adults differ in that matter. Moreover, it is known that in childhood, rMT measured by %-MSO is higher than in adolescence or in adulthood, and rMT among adolescents is comparable to that of adults (Säisänen et al. 2018). Based on a previous study in adolescence, the motor cortex has reached maturation as the cortical thickness appears stable (Wu et al. 2014), but has not yet started thinning due to aging.

We found a positive correlation between rMT and RD and MD of the extended hand knob area. On the contrary, we did not find an association between rMT and FA. There is no previous knowledge about the associations in question in adolescence. Our results are not completely consistent with the results of studies in adults; the evidence on the associations between white matter microstructure and rMT is mixed as Klöppel et al. found an inverse association between rMT and white matter FA across multiple brain regions (Klöppel et al. 2008), whereas Hübers et al. observed weak or no correlations between them (Hübers et al. 2012). Previous studies have shown that FA increases during adolescence (Barnea-Goraly et al. 2005; Schmithorst and Yuan 2010), whereas RD, MD, and AD decrease during maturation (Kumar et al. 2012; Qiu et al. 2008). Supporting these findings, we found an inverse correlation of age to RD, MD, and AD in the hand knob area on the right hemisphere, even though the age span of the participants was quite narrow. Myelination and axonal packing occur during adolescence and white matter maturation (Lebel and Deoni 2018) and therefore a decrease in RD in adolescence may reflect increased myelination (Song et al. 2005). Therefore, it is possible that continuously maturing white matter in adolescence partly explains the divergence between previous studies in adults and our study in adolescents. We found a correlation between rMT and MD, and there appears to be divergence between adults and adolescents, since in the adult population, a similar correlation has not been found (Hübers et al. 2012). The correlation between rMT and RD was novel; therefore, it is unclear whether adults and adolescents differ in this regard.

Although generally the motor cortex has reached maturation and rMT is comparable to that of adults in adolescence, there seem to be differences in the underlying causes of cortical excitability between adults and adolescents. Development of the neurotransmitter systems has been studied, and in adolescence, decreasing glutamate levels have been found at least in the prefrontal cortex (Perica et al. 2022). There is also evidence that GABAergic function increases in adolescence in the prefrontal cortex (Caballero et al. 2021). During development and cortical thinning, pruning of excitatory synapses occurs and proceeds at least in the prefrontal cortex to the third decade of life (Petanjek et al. 2011). It is possible that, during adolescence, changes in glutamatergic and GABAergic function also accompany cortical maturation in the motor cortex, affecting cortical excitability.

We observed a correlation between rMT and grey matter in the hand knob area and with white matter parameters in the extended hand knob area. This may be at least partially explained by the fact that the electric field distributes differently between grey and white matter (Opitz et al. 2011). Also, in previous studies in adults, correlations between FA and rMT were found in areas more widespread than the hand knob. In this study, we focused on the hand knob and extended hand knob areas in the motor cortex and did not consider other areas.

### Strengths and Limitations

A similar study has not been conducted on adolescents, and no entirely corresponding study on adults exists either. In this study, we used neuronavigation in the TMS procedure, therefore, it could be verified that the hotspot was located in the hand knob area. We were also able to take CCD into consideration. The analysis included a comprehensive set of white‑matter microstructural parameters: FA, MD, RD and AD, allowing a more detailed characterization of the underlying fiber architecture. A limitation of our study is the relatively small sample size that weakens statistical power in the analyses.

## Conclusion

In conclusion, we found a positive association between rMT and cortical thickness, as well as rMT and white matter parameters MD and RD in adolescence. The association with cortical thickness was found in the primary motor cortex, and with white matter parameters in the broader area of the primary motor cortex. Even though the grey matter of the motor cortex should have reached maturity in adolescence, it seems that still-evolving subcortical white matter affects cortical excitability, while brain function is still developing. This study sheds some light on the normal development and functions of the brain, which is necessary to understand the neurodevelopmental disorders occurring in adolescence. Further research is needed to comprehend the differences in the structural background of the cortical excitability across different age groups.

## Supplementary Information

Below is the link to the electronic supplementary material.

## Data Availability

Deidentified data will be available from the corresponding author on reasonable request.
