# Supplementary material for: The Associations of Grey Matter Thickness and White Matter Parameters on Corticospinal Excitability in Adolescence: A Navigated TMS Study
Source: Brain Topogr. 2026 Jul 18;39(5):81. doi: 10.1007/s10548-026-01236-0 (PMC13380597; doi:10.1007/s10548-026-01236-0)
Supplement: Supplementary file 1 [file 10548_2026_1236_MOESM1_ESM.docx]

**Supplementary Table 1**. The Pearson’s correlation coefficients (r) and P-values of the partial correlation analyses between resting motor threshold (rMT) and grey matter thickness (GM) of the hand knob area (HK) and the extended hand knob area (extHK) and white matter parameters fractional anisotropy (FA), mean diffusivity (MD), radial diffusivity (RD) and axial diffusivity (AD) of the hand knob (HK) and extended hand knob (ext HK) area. All analyses were performed separately on the left and right hemispheres.

|  |  | GM  HK | GM  ext HK | FA  HK | MD  HK | RD  HK | AD  HK | FA  ext HK | MD  ext HK | RD  ext HK | AD  ext HK |
| --- | --- | --- | --- | --- | --- | --- | --- | --- | --- | --- | --- |
| Left | r | 0.17  *0.11* | 0.17  *0.30* | 0.37  *0.021* | 0.15  *0.38* | 0.32  *0.50* | -0.24  *0.14* | 0.19  *0.28* | 0.45  *0.005* | 0.43  *0.006* | 0.34  *0.035* |
|  | P |  |  |  |  |  |  |  |  |  |  |
| Right | r | 0.31  *0.057* | 0.27  *0.10* | -0.13  *0.46* | 0.20  *0.22* | 0.21  *0.22* | 0.062  *0.71* | -0.020  *0.22* | 0.34  *0.036* | 0.38  *0.018* | 0.15  *0.36* |
|  | P |  |  |  |  |  |  |  |  |  |  |
